# The Fascinating World of Plant Non-Coding RNAs

**DOI:** 10.3390/ijms241210341

**Published:** 2023-06-19

**Authors:** Vesselin Baev, Andreas Gisel, Ivan Minkov

**Affiliations:** 1 Department of Plant Physiology and Molecular Biology, University of Plovdiv, 4000 Plovdiv, Bulgaria; 2 Institute of Biomedical Technologies (ITB), CNR, 70126 Bari, Italy; andreas.gisel@ba.itb.cnr.it; 3 Institute of Molecular Biology and Biotechnologies, 4108 Markovo, Bulgaria; minkov@plantgene.eu

Non-coding RNAs (ncRNAs), such as microRNAs (miRNAs), short interfering RNAs (siRNAs), and long non-coding RNAs (lncRNAs), have emerged as pivotal regulators within the plant kingdom. These previously unnoticed regulatory actors have been identified as integral components of a complex network, playing crucial roles in a wide range of pathways associated with plant development, plant health, and responses to environmental and disease-related factors. The advent of genome sequencing, particularly next-generation sequencing techniques and advanced bioinformatics tools, has revolutionized the process of discovering novel ncRNAs and elucidating their indispensable functions in plants. Nonetheless, further comprehensive investigations are warranted to elucidate the fundamental hotspots of the regulatory pathways in these plant processes governed by ncRNAs.

miRNAs and siRNAs play critical roles in various biological processes, including development, disease, and responding to environmental cues, by exerting their influence at the post-transcriptional level. Their ability to fine-tune gene expression and modulate cellular functions has attracted the attention of researchers seeking to unravel the intricate mechanisms underlying complex biological systems. Understanding the roles and functions of ncRNAs holds tremendous potential for advancing our knowledge of fundamental biological processes and has implications for the development of innovative plant health strategies.

In this Special Issue, we delve into the fascinating world of ncRNAs, exploring their importance and further shedding light on their involvement in shaping the complexity of life. Our goal is to provide up-to-date information about the non-coding RNA world in plants, further highlighting the role of these fascinating molecules and providing a platform for state-of-the-art research in this field.

Several authors in the current Special Issue have made significant contributions to the understanding of lncRNAs in plants. The research of Zhu et al. involved the exploration of both lncRNAs and mRNAs under cold stress conditions, leading to the profiling of numerous novel lncRNAs members [1]. lncRNAs as well as plant organogenesis and stress responses were also explored by Ran et al., characterized through a series of experiments, including cloning and sequencing a novel lncRNA molecule in poplar, elucidating its role in modulating the formation and development of adventitious roots [2].

Akpinar et al. explored putative lncRNAs in bread wheat and durum wheat varieties, emphasizing their role in defense against destructive pests. By analyzing and annotating lncRNAs from different wheat varieties, they uncovered the potential mechanisms underlying plant defense responses. The authors also observed a few putative lncRNAs that have perfect to near-perfect matches to organellar genomes, supporting the recent observations that organellar genomes may contribute to the non-coding transcript pool of the cell [3].

Further efforts have been made to understand the most famous ncRNA-miRNAs in plants. Luján-Soto et al. investigated the regulation of zma-MIR528a, a microRNA involved in nitrogen and auxin responses in plants. Their findings provide insights into the regulatory elements controlling the expression of this miRNA and contribute to the overall understanding of the miR528 regulome [4]. A common practice to understand the functional role of miRNAs is to combine different sequencing techniques and explore miRNA-mRNA regulatory networks. Mo et al. explored miRNA-mediated regulation in *M. micrantha* using transcriptome and small RNA sequencing. Their study provided valuable insights into the regulatory mechanisms underlying gene expression in *Mikania micrantha*, which causes huge ecological damage and economic losses worldwide due to its rapid growth and serious invasion [5]. Ding et al. also investigated the miRNA-mRNA regulatory networks, but in the phloem and developing xylem of poplar. Through integrated degradome and small RNA sequencing, they unraveled the interactions between miRNAs and target mRNAs, shedding new light on wood formation processes in poplar [6]. By integrating different omics approaches and methods, Pawełkowicz et al. studied the role of miRNAs in connection with the phenomenon of somaclonal variation, which occurs during plant in vitro culture [7].

This Special Issue also showcases the circular RNAs (circRNAs) that have been explored by Zhang et al., with a particular focus on poplar heterosis. Their study identified a substantial number of circRNAs in the poplar leaves of intergenic and exonic origins. They identified key circRNAs that may regulate poplar heterosis and revealed the molecular mechanism of heterosis formation in woody plants from a new perspective [8].

In addition to the research papers, several review studies in the field of miRNA roles in plant stress responses and medicinal plants [9,10], as well as bioinformatics approaches for identification of isomiRs [11], further extend and augment the knowledge in the ncRNAs literature, making them valuable addition to this Special Issue.

We expect that the Special Issue “The World of Plant Non-coding RNAs” will contribute up-to-date research on ncRNAs in plants, highlighting their importance and providing a platform for state-of-the-art research in this field. These findings reveal the diverse functions and regulatory mechanisms of ncRNAs in plants, advancing our understanding of fundamental biological processes, and offering potential applications in plant health and agricultural sciences.
